# Side‐Step Cutting Foot Biomechanics and Intrinsic Foot Muscle Sizes in People With Chronic Ankle Instability

**DOI:** 10.1002/jfa2.70222

**Published:** 2026-10-14

**Authors:** Jean‐Louis Peters‐Dickie, Christine Detrembleur, Sébastien Lobet, Kevin Deschamps

**Affiliations:** ^1^ Neuromusculoskeletal Lab (NMSK) Secteur des Sciences de la Santé Institut de Recherche Expérimentale et Clinique Université Catholique de Louvain (UCLouvain) Brussels Belgium; ^2^ Department of Rehabilitation Sciences Musculoskeletal Rehabilitation Research Group KU Leuven Bruges Belgium; ^3^ Division of Hematology Haemostasis and Thrombosis Unit Cliniques universitaires Saint‐Luc Université Catholique de Louvain (UCLouvain) Brussels Belgium; ^4^ Service d'ergothérapie et de kinésithérapie Cliniques universitaires Saint‐Luc Université Catholique de Louvain (UCLouvain) Brussels Belgium; ^5^ Division of Podiatry Haute Ecole Léonard De Vinci Brussels Belgium

**Keywords:** ankle sprain, change of direction, foot core, kinematics, kinetics, perceived instability, ultrasound imaging

## Abstract

**Introduction:**

Lateral ankle sprains commonly occur during directional changes and often lead to chronic ankle instability (CAI) associated with altered movement patterns, muscle weakness and atrophy. This study aimed to compare foot joint biomechanics during cutting among individuals with CAI, copers, and uninjured controls and to explore the relationship between intrinsic foot muscle sizes and functional status.

**Methods:**

Cross‐sectional study. Sixty‐nine recreationally active males (25.4 ± 4 years) were categorised by perceived instability and self‐reported function. Ankle and foot joint kinematics and kinetics were assessed using a multi‐segment foot model during 45° side‐step cutting at a mean approach speed of 3.5 m·s^−1^. Groups were compared using ANOVA or Kruskal–Wallis tests, and continuous biomechanical data were analysed using statistical parametric or non‐parametric mapping. Cross‐sectional area and thickness of the abductor hallucis and flexor digitorum brevis were recorded using ultrasound imaging. Muscle sizes were explored using general linear models, and correlations with questionnaire scores and physical examination outcomes were calculated.

**Results:**

Foot joint kinematics and kinetics did not significantly differ between the groups. Individuals with CAI exhibited lower peak posterior ground reaction force than controls (mean difference: −6.2% of body weight, 95% CI: −11.9 to −0.4, *p* = 0.03). Statistical parametric mapping revealed lower posterior ground reaction forces from 22% to 46% of stance in CAI versus controls (*p* < 0.05). Intrinsic foot muscle sizes showed weak, statistically significant negative correlations with perceived instability scores (*ρ* = −0.31 to −0.36) and self‐reported function scores (*ρ* = −0.26 to −0.33).

**Conclusion:**

Despite pronounced sensory‐perceptual impairments, individuals with CAI demonstrated few differences in foot joint biomechanics compared with copers and controls during a moderate‐speed anticipated cutting task. Reduced braking forces may reflect altered deceleration strategies. These findings suggest the importance of assessing deceleration abilities in people with CAI and indicate a potential role of foot muscles in CAI‐related functional outcomes.

**Trial Registration:**

ClinicalTrials.gov (NCT05815576)

## 1 Introduction

Lateral ankle sprain (LAS) is the most common injury in physically active individuals [1]. Approximately 40% of individuals who sustain a LAS develop chronic ankle instability (CAI) [1], whereas those remaining asymptomatic are termed copers [2]. CAI is defined by recurrent sprains, episodes of ankle giving way and perceived instability [3]. Individuals with CAI and copers have both sustained an initial ankle sprain, which is why direct comparisons between these populations are essential to understand the mechanisms underlying CAI development [2].

Hertel and Corbett's model summarises the impairments involved in CAI [4]. Motor‐behavioural impairments include altered movement patterns during locomotion and sporting tasks, balance deficits, and muscle weakness. Sensory‐perceptual impairments involve perceived instability, lower self‐reported function, and kinesiophobia. Pathomechanical impairments encompass structural deficits, including reduced lower leg muscle sizes and possibly reduced foot muscle sizes [4, 5].

Athletes participating in sports involving frequent changes of direction are at risk of LAS due to the vulnerable position of the ankle [6, 7]. Biomechanical studies found higher plantarflexion moment in early stance, lower ankle eversion moment at midstance [8], and greater ankle inversion during late stance of side cutting tasks in people with CAI compared to controls [9]. However, modelling the foot as a single segment may overestimate ankle kinematics and kinetics [10, 11], and multi‐segment foot models were therefore developed [10, 12, 13]. Foot joint kinetics during side‐step cutting remain uncharacterised in individuals with CAI compared with copers and uninjured controls. Kunugi et al. found greater hindfoot external rotation in late stance in CAI with severe laxity versus CAI without laxity [14], and similar findings when comparing copers with and without laxity [15]. In addition, other studies found sagittal and frontal plane kinematic differences at the hindfoot and forefoot during running between CAI and controls [16, 17]. The arthrokinematic and osteokinematic restrictions associated with CAI [4] likely impair foot joint kinetics, as has been observed in other foot joint disorders [18, 19, 20]. Therefore, investigating foot joint biomechanics during side‐step cutting may provide insights into functional alterations and assist in developing rehabilitation strategies for individuals with LAS and CAI.

Foot function is supported by both extrinsic and intrinsic foot muscles (IFMs), the latter originating within the foot [21, 22]. The IFMs, with the abductor hallucis and flexor digitorum brevis being among the largest, attenuate foot impact and support push‐off during locomotion [23, 24] and also have proprioceptive roles [25].

Ultrasound imaging can reliably assess IFM sizes [26, 27], whereas dynamometry provides a more direct assessment of hallux and toe flexion strength [28, 29]. Although previous research demonstrated reduced ankle strength in multiple directions among individuals with CAI [30], few studies have examined muscle sizes and strength more distally at the foot level [5, 31]. Feger et al. reported smaller IFM sizes in individuals with CAI compared to controls, in a small sample of ten participants [5]. Fraser et al. observed deficits in hallux and toe flexion strength in CAI relative to both copers and uninjured controls, but also found that abductor hallucis, flexor digitorum brevis, quadratus plantae and flexor hallucis brevis cross‐sectional areas were more strongly associated with sex and body mass index (BMI) than with CAI itself [31]. Clarifying whether reduced IFM sizes and foot strength are characteristics of CAI is crucial for rehabilitation, as these parameters are modifiable through targeted interventions [32].

The aim of this study was to address specific domains highlighted in the model of Hertel and Corbett, particularly movement patterns and muscle sizes [4]. To our knowledge, this is the first study to use a kinetic multi‐segment foot model to compare foot joint kinematics and kinetics during side‐step cutting among individuals with CAI, copers, and uninjured controls. The primary objective of this study was to assess ankle and foot joint kinematics and kinetics during side‐step cutting among individuals with CAI, copers and uninjured controls. We hypothesised that participants with CAI would exhibit significantly lower ankle sagittal plane range of motion (ROM) in early stance than copers and uninjured controls. We also hypothesised that the CAI group would demonstrate significantly lower ankle and Chopart joint power absorption during impact and active phases, lower power generation during the propulsive phase, and differences in joint moments compared with both other groups. The secondary, exploratory objective was to compare abductor hallucis and flexor digitorum brevis muscle sizes across the three groups of participants, while controlling for BMI, and to examine their correlations with patient‐reported outcomes and physical examination outcomes.

## 2 Materials and Methods

### 2.1 Study Design

This cross‐sectional study followed the STROBE guidelines [33]. The trial procedures were prospectively registered with ClinicalTrials.gov (NCT05815576).

### 2.2 Participants

Physically active males aged 18–44 were recruited between January 2023 and February 2025 from universities and high schools (students and staff members) in Belgium. Participants were considered physically active if they reported ≥ 1 hour per week of physical exertion. The study followed the Declaration of Helsinki and was approved by the ethical committee of the University Hospitals Leuven. Written informed consent was obtained before data collection.

An a priori power analysis (GPower 3.1) [34] indicated 21 participants per group for the main hypothesis of this study, with power = 0.85 and *α* = 0.05. The calculation used previously published data comparing ankle joint ROM between males with CAI and controls, collected during running in the same laboratory, resulting in Cohen's *f* = 0.43 [35].

Inclusion criteria for CAI were (1) a first substantial LAS that occurred ≥ 1 year ago, (2) the last substantial LAS occurred ≥ 3 months ago, (3) two or more recurrent sprains, giving way episodes, or feelings of instability in the last six months, and (4) perceived ankle instability confirmed by a Cumberland Ankle Instability Tool (CAIT) score ≤ 24 [3, 4, 36]. Copers experienced their most recent substantial LAS ≥ 1 year before enrolment. Copers and controls had CAIT ≥ 28 [2, 4], and Foot and Ankle Ability Measure (FAAM) subscales ≥ 95% [4, 37]. A “substantial LAS” was defined as severe enough to interrupt weight‐bearing physical activities for at least one day [3].

Exclusion criteria: Any systemic, orthopaedic or neurological diseases, major lower‐limb deformities, a bilateral leg length difference > 3 cm, ankle surgery, and BMI > 30 kg·m^−2^ (to ensure accurate marker tracking during side‐step cutting).

### 2.3 Measurement Set‐Up

All measurements were conducted at the Clinical Motion Analysis Laboratory (Leuven, Belgium). Passive reflective markers (9.5‐ and 12‐mm diameter) were placed on anatomical landmarks according to the *IOR‐4segment‐model‐1* (i.e., the Rizzoli foot model and Plug‐in Gait lower body model) [12, 38]. Markers were tracked by 10 T‐10 infrared cameras (100 Hz, Vicon, Oxford Metrics, UK). A pressure plate (2.8 sensors·cm^−2^, 200 Hz, RsScan International, Belgium) was positioned on top of a force plate (1000 Hz, Advanced Mechanical Technology, USA) both embedded within the walkway. A 3D box (Materialise Motion, Paal, Belgium) ensured signal synchronisation.

### 2.4 Measurement Protocol and Processing

Demographics, sprain history [3], sporting habits, 6‐item Foot Posture Index (FPI‐6) [39, 40] and sit‐to‐stand navicular drop were collected. Navicular drop was measured as the difference in navicular tuberosity height between sitting (leg vertical) and standing (feet hip‐width apart) using a calliper (resolution 0.02 mm). The navicular tuberosity was first marked with a pen to ensure consistent measurements [41]. Dynamic balance was assessed with the modified Star Excursion Balance Test in the anterior, posterolateral and posteromedial directions, with three trials per direction. The maximal distance in each direction was normalised to leg length and used to calculate a composite score [42].

Hallux and toe flexion strengths were measured in long sitting, with the ankle maximally plantarflexed, using a hand‐held dynamometer (microFET2, Biometrics, France) [29]. Participants pressed maximally for 5 seconds, repeating trials until there was less than 20% variability across the last three trials, and the mean of the two highest values was analysed.

Participants were instructed to perform an anticipated barefoot 45° side‐step cutting task at 3.3 m·s^−1^ (± 10%). After two practice trials and static calibration, five valid trials were collected. A trial was considered valid when the prescribed speed and cutting angle were achieved. Foot‐strike and toe‐off events were manually identified using Nexus 2.12 software (Vicon, Oxford Metrics, UK) and marker trajectories were filtered using a fifth‐order Woltring spline filter, with the predicted mean‐square‐error parameter set to 10 mm^2^. No additional filtering was applied to the marker trajectories, force‐plate data, or pressure data. Data were then processed using an in‐house MATLAB program (ACEP_Manager, Leuven, Belgium). Trials were processed according to the *IOR‐4segment‐model‐1*, which distinguishes the shank, hindfoot, midfoot, metatarsus and hallux segments, separated by the ankle, Chopart, Lisfranc, and hallux metatarsophalangeal joints [12, 38, 43].

Joint angular positions and velocities were determined in the sagittal, frontal and transverse planes. Subsequently, internal joint moments were calculated by an inverse dynamics approach (Newton‐Euler equations) with vertical ground reaction force (GRF) distributed according to foot pressure distribution beneath foot segments [12]. Joint power was computed as moment×angularvelocity. Curves from each trial and each outcome were visually inspected for data quality or processing artefacts. Trials showing evident abnormalities were excluded from the analyses.

Data were normalised to 101 points over the stance phase, and the mean curve of each participant was further used in the analyses. Foot strike index [44], spatiotemporal parameters, joint ROM, positive and negative work, and the peaks in joint velocity, moment, power and GRF were computed. More precisely, ROM was calculated within the impact (0%–6% of stance), active (7%–45%) and propulsive (46%–100%) subphases of stance [35]. Kinetic outcomes were normalised to body weight.

Ultrasound imaging (Telemed Echoblaster, 10‐MHz linear transducer) was used to obtain images of abductor hallucis and flexor digitorum brevis following a previously described protocol [26]. Muscle cross‐sectional area and thickness were measured using ImageJ software. Previous studies have generally reported good‐to‐excellent test–retest reliability for ultrasound measurements of IFM morphology [45]. Using the same protocol as in the present study, with repeated image acquisition and measurement, test–retest reliability was moderate to good for these outcomes, with intraclass correlation coefficients ranging from 0.51 to 0.88 [26].

### 2.5 Statistics

A limited number of zero‐dimensional outcomes were designated as primary and informed the a priori sample‐size calculation. Other zero‐dimensional outcomes and one‐dimensional waveform analyses were secondary, whereas analyses of intrinsic foot muscle morphology and its associations with clinical outcomes were exploratory.

The significance level was set at *α* = 0.05. Zero‐dimensional data were analysed using SPSS v27 (IBM, USA). Categorical data were compared with Chi‐squared tests. Normality of quantitative data was assessed using the Shapiro–Wilk test, and homogeneity of variances was assessed through Levene's test. Normally distributed data were analysed using one‐way ANOVA (with or without Welch correction) followed by post hoc t‐tests. Non‐normally distributed data were compared using Kruskal–Wallis tests followed by Mann–Whitney post hoc tests. Post hoc *p*‐values and analyses related to ROM were corrected using Bonferroni corrections, while no additional correction was applied across all biomechanical outcomes because they represented distinct joints, planes, and constructs, and such a global correction would have been highly conservative for the secondary analyses. Mean differences and median differences were used as effect size estimates. Cross‐correlation joint coupling coefficients were interpreted as weak (< 0.3), moderate (0.3–0.69), and strong (≥ 0.7) [46].

Kinematic and kinetic curves were analysed as preplanned secondary outcomes using one‐dimensional Statistical Parametric Mapping or non‐Parametric Mapping (SPM or SnPM) in MATLAB R2022a with the open‐source spm1d package (spm1d.org) [47, 48]. Normality of the ANOVA residual field was assessed. Then, a one‐way between‐group SPM{F} ANOVA was used when normality was not rejected; otherwise, the corresponding SnPM{F} ANOVA was used. Significant omnibus effects were followed by Bonferroni‐corrected pairwise two‐sample SPM or SnPM t‐tests.

IFM sizes were analysed exploratively using general linear models with BMI as a covariate, followed by correlation analyses between IFM sizes, questionnaire scores, balance, foot posture, foot mobility, and strength, reporting Pearson (*r*) or Spearman (*ρ*) coefficients depending on normality. Coefficients were interpreted as low (0.3–0.49), moderate (< 0.7), high (< 0.9), and very high (≥ 0.9) [49].

## 3 Results

### 3.1 Participants

A total of 22 participants with CAI, 24 copers and 23 controls were included (Figure 1). Demographics are displayed in Table 1, while ankle sprain history and rehabilitation details are provided in Supporting Information S1: Table S1. Participants with CAI reported lower ankle function than the two other groups, with median differences of −5 percentage points for the Activities of Daily Living subscale and −12.5 percentage points for the Sports subscale (*p* < 0.001). Post hoc tests are detailed in Supporting Information S1: Table S2.

**FIGURE 1:**
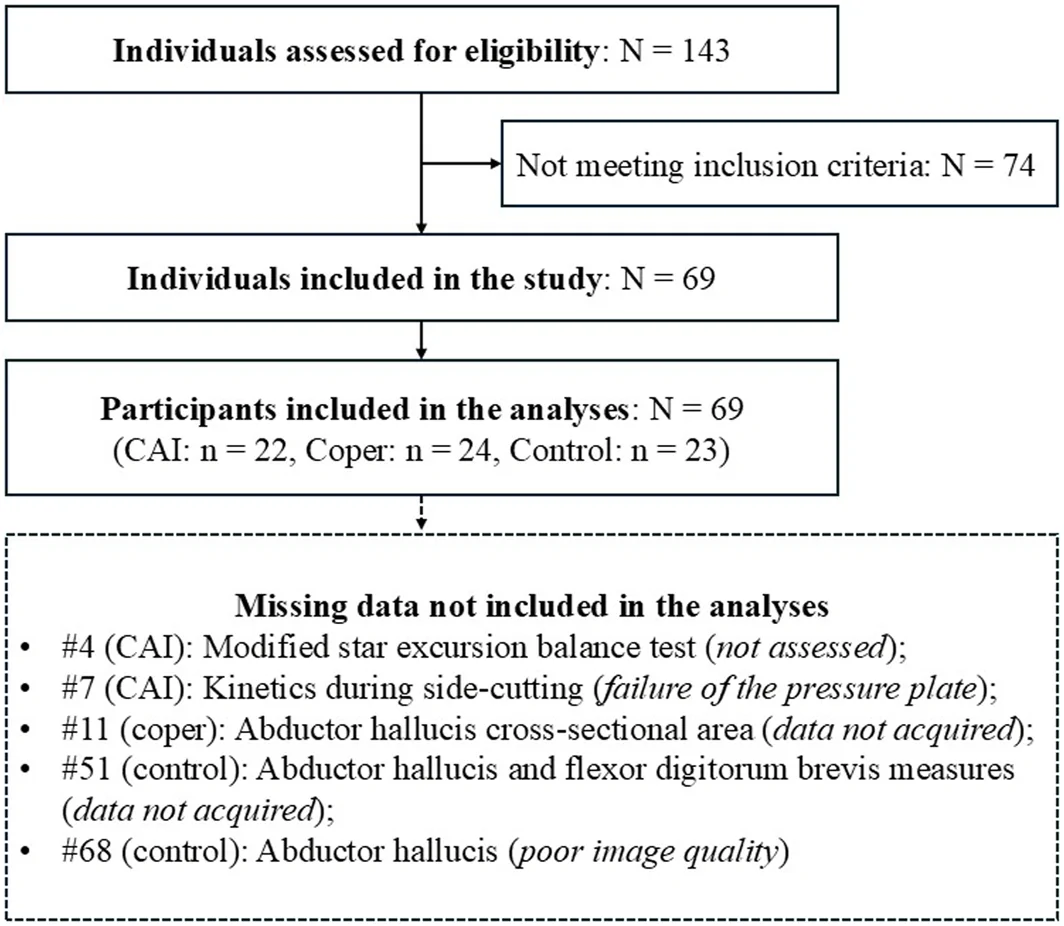
Flow chart of the participants included in the study. CAI: participants with chronic ankle instability, *N*: number of participants, *n*: number of participants in a subgroup, #: participant identification number. Missing data are detailed at the bottom of the figure, including the reason for missingness.

**TABLE 1:** Demographics and self‐reported outcome measures.

Outcome	CAI (*n* = 22)^a^	Coper (*n* = 24)^b^	Control (*n* = 23)^c^	*p*‐value
Age (years)†	25.8 (5.4)	25.9 (3)	23.9 (4)	0.091
Height (cm)‡	180.4 (6.9)	180.7 (6)	179.3 (6.9)	0.747
Weight (kg)†	77.8 (9.7)	77.5 (8.7)	72 (6.7)	0.039*
BMI (kg·m^−2^)‡	23.9 (2.5)	23.8 (2.9)	22.5 (2.3)	0.095
Shoe size (European)‡	43.3 (1.6)	43.5 (1.4)	43 (1.6)	0.516
Dominant side (*n*)	3 left/19 right	4 left/20 right	4 left/19 right	0.936
Affected side (*n*)	13 dominant/9 non‐dominant	17 dominant/7 non‐dominant	—	0.30
Weekly physical activity (hours·week^−1^)‡	5.5 (4.4)	5.5 (3.4)	5.1 (2.6)	0.936
CAIT (/30)‡	17.5 (4.6)^b,c^	29.2 (1.2)^a^	29.6 (0.9)^a^	< 0.001*
FAAM ADL subscale (%)‡	90.3 (9.8)^b,c^	99.7 (0.7)^a^	99.9 (0.3)^a^	< 0.001*
FAAM sports subscale (%)‡	81.3 (16.5)^b,c^	99.3 (2.1)^a^	99.3 (1.9)^a^	< 0.001*
FABQ physical activity (/24)‡	7.4 (5.6)	5.4 (5.5)	4.3 (4.4)	0.127
FABQ work (/42)‡	3.3 (5.1)	1.1 (2.7)	1.4 (3)	0.102
FABQ total (/66)‡	10.7 (9.5)	6.5 (6.9)	5.7 (5.7)	0.089

*Note:* Data are presented as mean (standard deviation). ADL (activities of daily living), CAI (chronic ankle instability), CAIT (Cumberland ankle instability tool), FAAM (foot and ankle ability measure), FABQ (fear avoidance belief questionnaire), *n* (number of participants), † (normality not rejected), ‡ (normality rejected), * (statistically significant); superscript letters indicate significant group differences during post‐hoc tests (*p* < 0.001).

### 3.2 Physical Examination

Individuals with CAI and copers had lower normalised hallux flexion strength than controls, with mean differences of −0.25 N·kg^−1^ (95% CI: −0.50 to 0.00, *p* = 0.050) and −0.28 N·kg^−1^ (95% CI: −0.52 to −0.03, *p* = 0.02), respectively (Table 2).

**TABLE 2:** Outcomes from the physical examination.

Outcomes	CAI^a^	Coper^b^	Control^c^	*p*‐value
mSEBT anterior (proportion of leg length)‡	0.9 (0.06)	0.91 (0.08)	0.91 (0.07)	0.589
mSEBT posteromedial (proportion of leg length)†	0.91 (0.1)	0.96 (0.09)	0.96 (0.08)	0.135
mSEBT posterolateral (proportion of leg length)†	0.87 (0.11)	0.93 (0.08)	0.93 (0.08)	0.073
mSEBT composite (proportion of leg length)†	0.9 (0.08)	0.93 (0.06)	0.93 (0.06)	0.105
Hallux flexion strength (N·kg^−1^)†	1.09 (0.32)^c^	1.07 (0.34)^c^	1.35 (0.37)^a,b^	0.014*
Other toes flexion strength (N·kg^−1^)†	1.04 (0.31)	1.13 (0.29)	1.22 (0.33)	0.178
FPI‐6 (score −12 to +12)‡	4.5 (2.8)	3.2 (3.5)	3.5 (4.3)	0.455
Navicular drop (cm)‡	0.63 (0.29)	0.67 (0.27)	0.64 (0.27)	0.623

*Note:* Data are presented as mean (standard deviation). CAI (chronic ankle instability), mSEBT (modified star excursion balance test), † (normality not rejected), ‡ (normality rejected), * (statistically significant); superscript letters indicate the groups that differed significantly during post‐hoc tests.

### 3.3 Side‐Step Cutting Task

Participants performed side‐step cutting with a mean approach speed of 3.45 m·s^−1^, close to the target speed of 3.3 m·s^−1^ (± 10%). Sagittal‐plane ankle and foot ROM, moments, power, and work did not differ between groups, contrasting with our hypotheses (Table 3).

**TABLE 3:** Ankle and foot joint kinematics and kinetics during side‐step cutting.

Outcomes	CAI	Coper	Control	*p*‐value
ANKLE				
PF/DF ROM: P1 (°)‡	4.7 (1.8)	5 (2.1)	4.3 (1.8)	1
PF/DF ROM: P2 (°)†	20.4 (3.1)	21.3 (3.4)	20.6 (2.9)	1
PF/DF ROM: P3 (°)†	38.8 (7.5)	41 (4.1)	40.9 (4.7)	1
Peak PF moment (N·m·kg^−1^)†	2.48 (0.34)	2.57 (0.42)	2.65 (0.4)	0.33
Peak gen. power (W·kg^−1^)†	12.3 (3.25)	13.66 (2.35)	13.93 (2.5)	0.117
Peak abs. power (W·kg^−1^)‡	4.71 (2.15)	5.53 (1.89)	5.88 (3.15)	0.245
Pos. work (J·kg^−1^)‡	1.04 (0.25)	1.11 (0.19)	1.17 (0.18)	0.45
Neg. work (J·kg^−1^)†	0.42 (0.17)	0.51 (0.19)	0.49 (0.2)	0.228
CHOPART				
PF/DF ROM: P1 (°)†	2.9 (1.8)	3.2 (2)	2.7 (2)	1
PF/DF ROM: P2 (°)‡	6.2 (1.3)	6.4 (2.4)	6.9 (2.5)	1
PF/DF ROM: P3 (°)†	17 (3.8)	17.5 (5.4)	18.3 (4.2)	1
Peak PF moment (N·m·kg^−1^)†	1.83 (0.29)	1.91 (0.31)	1.95 (0.3)	0.45
Peak gen. power (W·kg^−1^)†	4.24 (1.22)	4.44 (1.97)	4.74 (1.09)	0.538
Peak abs. power (W·kg^−1^)‡	2.14 (1)	2.23 (1.03)	2.49 (1.16)	0.527
Pos. work (J·kg^−1^)†	0.23 (0.05)	0.23 (0.09)	0.26 (0.07)	0.354
Neg. work (J·kg^−1^)‡	0.17 (0.05)	0.18 (0.06)	0.19 (0.05)	0.623
LISFRANC				
PF/DF ROM: P1 (°)‡	1.3 (1)	1.4 (1.1)	1.9 (1.1)	0.46
PF/DF ROM: P2 (°)‡	2.7 (1.8)	2.2 (1.4)	3.1 (1.5)	0.19
PF/DF ROM: P3 (°)‡	8 (3.1)	7.8 (2.6)	7.5 (2.6)	1
Peak PF moment (N·m·kg^−1^)‡	0.78 (0.22)	0.8 (0.22)	0.83 (0.18)	0.71
Peak gen. power (W·kg^−1^)‡	2.4 (1.16)	2.31 (0.68)	1.99 (0.64)	0.395
Peak abs. power (W·kg^−1^)‡	0.4 (0.29)	0.31 (0.16)	0.46 (0.31)	0.189
Pos. work (J·kg^−1^)†	0.15 (0.06)	0.12 (0.04)	0.12 (0.03)	0.137
Neg. work (J·kg^−1^)‡	0.025 (0.015)	0.021 (0.011)	0.027 (0.014)	0.318
HALLUX				
PF/DF ROM: P1 (°)‡	6.9 (5.3)	5.9 (3.6)	5.4 (2.7)	1
PF/DF ROM: P2 (°)‡	7.1 (3.7)	6.4 (2.4)	7.5 (4.2)	1
PF/DF ROM: P3 (°)†	39.1 (7.6)	42.2 (5.6)	41.3 (6.5)	0.83
Peak PF moment (N·m·kg^−1^)‡	0.26 (0.08)	0.24 (0.08)	0.28 (0.09)	0.36
Peak gen. power (W·kg^−1^)‡	0.41 (0.22)	0.41 (0.32)	0.45 (0.21)	0.579
Peak abs. power (W·kg^−1^)‡	2.56 (1)	2.58 (0.89)	2.69 (0.84)	0.609
Pos. work (J·kg^−1^)‡	0.014 (0.007)	0.013 (0.011)	0.016 (0.007)	0.114
Neg. work (J·kg^−1^)†	0.15 (0.05)	0.14 (0.06)	0.16 (0.05)	0.527

*Note:* Data are presented as mean (standard deviation). Abs. (absorption), DF (dorsiflexion), gen. (generation), neg. (negative), P1 (impact subphase), P2 (active subphase), P3 (propulsive subphase), PF (plantarflexion), pos. (positive), ROM (range of motion), † (normality not rejected), ‡ (normality rejected). *p*‐values were corrected to account for the number of subphases for ROM, by multiplying the value by 3.

Participants with CAI had lower posterior peak GRFs than controls, with a mean difference of −6.2% of body weight (95% CI: −11.9 to −0.4, *p* = 0.03; Table 4). SPM similarly identified lower posterior GRFs in the CAI group from 22% to 46% of stance (*p* < 0.05; Figure 2).

**TABLE 4:** Peak ground reaction forces during side‐step cutting.

Outcomes	CAI^1^	Coper^2^	Control^3^	*p*‐value
Vertical (% body weight)†	216.4 (23.5)	222.5 (29.2)	230.9 (26.1)	0.20
Medial (% body weight)†	49 (9.1)	51.4 (5.7)	51.2 (8)	0.53
Posterior (% body weight)†	32 (7.9)^3^	33 (6.9)	38.2 (8.4)^1^	0.021*
Anterior (% body weight)†	16.8 (4.1)	18.8 (3.6)	16.7 (4.8)	0.18

*Note:* Data are presented as mean nd standard deviation. CAI (chronic ankle instability), † (normality not rejected), * (statistically significant); superscript numbers indicate the groups that differed significantly during post‐hoc tests.

**FIGURE 2:**
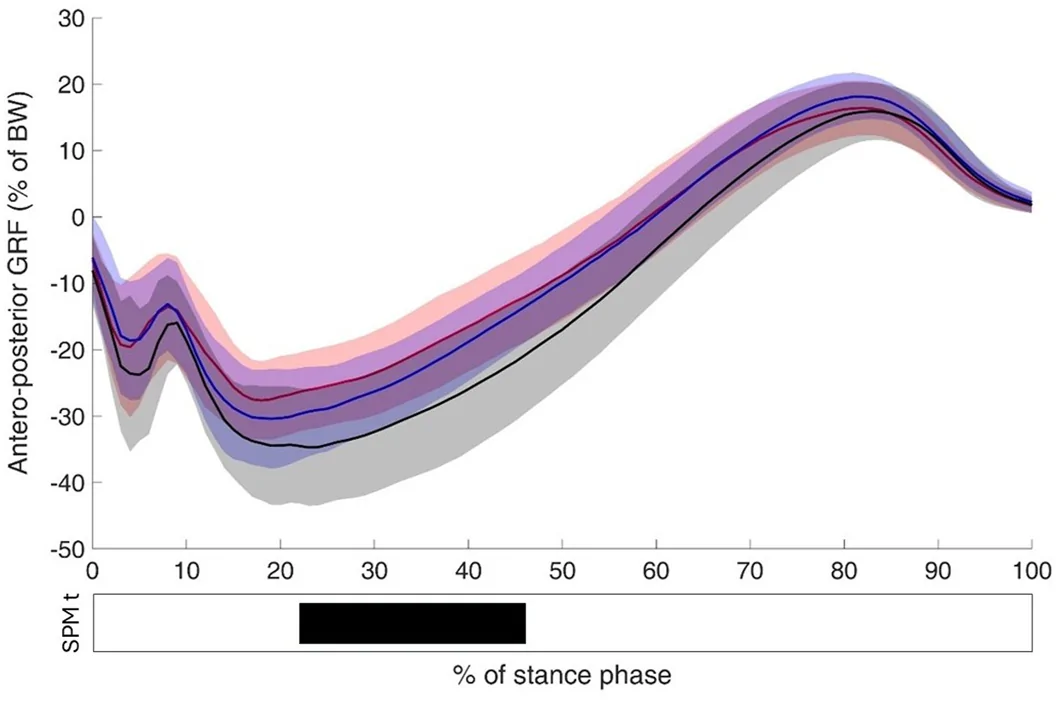
Mean antero‐posterior ground reaction forces during the stance phase in individuals with chronic ankle instability (red), copers (blue), and controls (black), with shaded areas representing standard deviations. The critical SPM ANOVA threshold was exceeded from 22% to 56% of stance (F* = 6.55, *p* < 0.001). Post hoc testing showed significantly lower posterior ground reaction forces in individuals with chronic ankle instability than in controls from 22% to 46% of stance (critical t* = ± 3.61, *p* < 0.05), as indicated by the horizontal significance bar below the graph. BW: body weight, GRF: ground reaction force, SPM: statistical parametric mapping.

No between‐group differences were found for spatiotemporal parameters or frontal‐ and transverse‐plane joint outcomes, and cross‐correlation coefficients were weak (|*r*| < 0.3; Supporting Information S1: Tables S3–S5).

### 3.4 IFM Sizes

IFM sizes were analysed using general linear models including BMI and group (Table 5), with descriptive values provided in Supporting Information S1: Table S6 and visual information displayed in Figure S1. BMI was positively associated with the flexor digitorum brevis cross‐sectional area (overall model *p* = 0.03, adjusted *R* ^2^ = 0.094; BMI *p* = 0.02). No significant group effect was identified for any IFM measure.

**TABLE 5:** General linear models related to foot muscle sizes.

Model features	Model parameters	B	Partial η^2^	*p*
Abductor hallucis CSA	Intercept	1.6	—	—
*R* ^2^ = 0.07	BMI	0.03	0.02	0.23
Adjusted *R* ^2^ = 0.03	Group	—	0.04	0.26
*p* = 0.19	Group = CAI	0.24	—	—
	Group = Coper	0.02	—	—
Abductor hallucis thickness	Intercept	0.75	—	—
*R* ^2^ = 0.15	BMI	0.02	0.05	0.08
Adjusted *R* ^2^ = 0.10	Group	—	0.08	0.08
*p *= 0.018*	Group = CAI	0.15	—	—
	Group = Coper	0.07	—	—
Flexor digitorum brevis CSA	Intercept	1.23	—	—
*R* ^2^ = 0.14	BMI	0.05	0.08	0.020*
Adjusted *R* ^2^ = 0.09	Group	—	0.04	0.3
*p* = 0.025*	Group = CAI	0.18	—	—
	Group = Coper	0.04	—	—
Flexor digitorum brevis thickness	Intercept	0.74	—	—
*R* ^2^ = 0.07	BMI	0.02	0.06	0.049*
Adjusted *R* ^2^ = 0.03	Group	—	0.04	0.88
*p* = 0.18	Group = CAI	0.02	—	—
	Group = Coper	0.01	—	—

*Note:* B (coefficient of regression), BMI (body mass index), CAI (chronic ankle instability), *p* (*p*‐value), *R* ^2^ (goodness of fit of the model), *η* ^2^ (eta‐squared), * (statistically significant). A model was interpreted if the *p*‐value of the model was statistically significant. Adjusted *R* ^2^ was used to estimate the proportion of variance in muscle size explained by the model.

Cross‐sectional area and thickness were strongly correlated for abductor hallucis (*r* = 0.72) and moderately correlated with flexor digitorum brevis (*r* = 0.51). Weak but statistically significant correlations were found between larger IFM measures and lower CAIT and FAAM scores (*ρ* = −0.26 to −0.36), while abductor hallucis measures were weakly positively correlated with foot posture (*ρ* = 0.32–0.33; Supporting Information S1: Table S7 and Figure S2).

## 4 Discussion

This was the first study to compare multi‐segment foot joint kinematics and kinetics during side‐step cutting among individuals with CAI, copers, and uninjured controls. Contrary to our hypotheses, no significant sagittal plane kinematic differences and only limited kinetic discrepancies were found. These findings suggest that foot joint biomechanics may not clearly differentiate the groups under the anticipated, moderate‐speed conditions examined.

CAI is a multifactorial condition resulting from the interaction of various impairments [4]. In our study, individuals with CAI reported higher levels of perceived instability and self‐reported disability compared with the two other groups. In their systematic review, Houston et al. reported Hedges' *g* effect sizes ranging from 1.8 to 3.3 when comparing CAIT scores between individuals with CAI and controls, and from 1 to 3.3 for the FAAM subscales [50]. Although we used median differences due to skewed data distributions, our results corresponded to Hedges' *g* values of 3.6 for CAIT and 1.4 to 1.6 for FAAM subscales when comparing CAI to controls. Comparable effect sizes would be obtained when comparing CAI to copers. Despite these sensory‐perceptual impairments, individuals with CAI showed few differences in motor‐behavioural factors (cutting biomechanics, balance, strength) or structural measures (IFM sizes). This highlights the need for clinicians to assess and target multiple CAI domains in rehabilitation [51, 52].

A qualitative comparison of kinematic curves confirmed that the three groups exhibited similar movement patterns. To our knowledge, only one previous study has compared cutting kinematics between individuals with CAI and controls—specifically in female football players—and reported no between‐group differences [53]. During running, although some evidence suggests altered foot joint movement patterns in individuals with CAI [16, 17], others have reported no significant differences compared to uninjured controls [54, 55]. Qualitatively, some apparent kinetic differences were observed across the groups. Specifically, individuals with CAI tended to exhibit lower ankle power generation in late stance and lower Chopart plantarflexion moment compared with the two other groups. Additionally, both individuals with CAI and copers appeared to show less Chopart power generation in late stance, as well as lower hallux power absorption and hallux dorsiflexion moment compared to controls. As this was the first study to investigate foot joint kinetics across these groups, direct comparison with existing literature is limited.

Conventional joint‐level analyses may not fully capture how movement is coordinated between adjacent segments, including the relative timing and contribution of each segment. During walking, individuals with CAI, copers, and controls have shown phase‐dependent coordination differences [56]. Similarly, during running, individuals with CAI and copers have shown different foot–shank coordination patterns and coordination variability compared with controls [57], while intra‐foot analyses identified distinct rearfoot–midfoot and midfoot–forefoot coordination patterns between CAI and copers [58]. Future studies should determine whether similar coordination patterns occur during side‐step cutting and whether they reveal group differences not detected through individual joint kinematics.

SPM analysis identified lower posterior GRFs during the first half of stance in CAI compared to controls, along with lower peak posterior GRFs in CAI. To our knowledge, these findings of lower posterior braking forces during changes in direction are unique under anticipated 45° cutting conditions. Previous studies either did not report antero‐posterior GRFs [14, 15, 59] or found no between‐group differences during a 45° task [53]. Previous landing‐cutting studies have also reported reduced ankle energy absorption and generation together with greater knee or hip contributions in individuals with CAI, supporting a possible distal‐to‐proximal redistribution of mechanical work [60, 61]. A comparable compensatory strategy may have reduced the contribution of the ankle‐foot complex to braking in the present study. However, posterior GRF also depends on whole‐body deceleration, contact time, approach and exit velocities, centre‐of‐mass trajectory, and the executed cutting angle [62]. Because these variables, proximal joint mechanics, and muscle activation were not assessed, the underlying mechanism remains uncertain. Future studies should assess whole‐body deceleration more comprehensively, including approach and exit velocities, braking impulses, contact times, and centre‐of‐mass trajectory, alongside foot and proximal joint biomechanics. From a performance perspective, reduced braking forces may impair directional change efficiency, as greater posterior braking forces have been associated with faster cutting performance in uninjured athletes [63]. These findings suggest that individuals with CAI may adopt altered deceleration strategies during changes in direction. Although this interpretation warrants further investigation, these findings reinforce the potential relevance of assessing sport‐specific agility and cutting performance in individuals with CAI [64].

Models including BMI and group accounted for approximately 10% of the variance in abductor hallucis thickness and flexor digitorum brevis cross‐sectional area, indicating limited explanatory value, and group was not a significant predictor. These findings are consistent with recent studies showing that CAI, coper, or control group status has little influence on IFM sizes [31, 65]. Similarly, Fraser et al. explained 13%–24% of the variance in IFM sizes using models with sex, BMI, group, foot posture, and foot mobility, leaving a substantial proportion unexplained [31]. Weak inverse correlations (*ρ* = −0.27 to −0.36) associated larger IFM measures with greater perceived instability and poorer self‐reported function. Given their small magnitude, these correlations should not be interpreted as clinically meaningful. In addition, the direction of these associations was counterintuitive. Further studies are required before conclusions can be drawn regarding the relationship between IFM morphology and patient‐reported outcomes.

We also investigated foot strength using dynamometry and found lower normalised hallux flexion strength in CAI and copers versus controls, and relatively similar toe flexion strength across groups. These results are partially in agreement with those of Fraser et al., who reported lower normalised hallux and toe flexion strengths in CAI compared with copers and controls [31]. Hallux flexion and toe flexion strengths have been associated with functional abilities, such as static and dynamic balance performance [66, 67]. Only a couple of studies have investigated associations between foot strength and change of direction performance, with conflicting findings [68, 69]. The presence of lower hallux flexion strength despite relatively similar IFM sizes across groups suggests that muscle morphology alone may not fully capture functional impairments. This discrepancy may reflect alterations in neuromuscular control, potentially including neuromuscular inhibition in CAI [4]. IFM function should therefore be further explored in CAI, as identifying impairments in muscle activation or force‐generating capacity would provide a stronger rationale for evaluating targeted IFM interventions [32].

Personal and task‐related factors influence kinematics, kinetics, and GRFs during a change of direction. First, individuals with CAI may present with heterogeneous alterations [4, 70]. In our study, perceived instability served as the criterion to distinguish between CAI and the two other groups. Mechanical ankle joint laxity is a potential confounding factor that influences side‐step cutting biomechanics, as consistently reported in two previous studies [14, 15]. In addition, our participants with CAI reported few alterations in self‐reported function, many practised sports involving changes in direction, and the majority pursued rehabilitation. These factors could explain the absence of biomechanical differences between groups. Second, task‐related factors such as approach speed, cutting angle, footwear, and anticipation may have influenced the outcomes, as these factors affect cutting mechanics, braking demands, and muscle activation [62]. Takeuchi and Anan found differences in horizontal GRFs between individuals with CAI and controls during 90° and 135° cutting, but not during a 45° task performed at a self‐selected comfortable speed [53]. The anticipated and submaximal nature of the present task may therefore have reduced its sensitivity to detect between‐group differences. Similar joint‐level kinematics do not necessarily indicate identical motor control. During landing‐cutting, individuals with CAI and controls have shown similar overall muscle‐synergy complexity but different muscle‐specific weighting [71], while planned landing has been shown to mask CAI‐related differences in ankle orientation and peroneal activation that emerged under unplanned conditions [72]. Anticipation may therefore have allowed feedforward preparation and compensatory motor strategies that maintained a broadly similar foot movement pattern. Anticipation also modifies GRFs, joint mechanics, and muscle activation during side cutting in uninjured athletes [73]. Competitive sporting situations also involve greater speed, fatigue, environmental uncertainty, and perceptual and decision‐making demands [74]. Future studies should therefore examine faster, larger‐angle, and unanticipated cutting tasks incorporating sport‐relevant visual or auditory cues.

Beyond manipulating task demands, future research may also benefit from more individualised assessment of foot loading. High‐resolution plantar‐pressure analyses can provide spatial information across the entire plantar surface and allow group‐ and individual‐level loading patterns to be compared with reference distributions [75, 76]. Direct plantar shear measurements integrated with multi‐segment foot models could further clarify how horizontal braking and propulsive forces are distributed across foot segments [77]. Personalised finite‐element models could complement these external measurements by estimating internal bone and soft‐tissue stresses. Such an approach has already identified distinct internal loading patterns among individuals with CAI, copers, and controls during cutting [78]. Together, these complementary approaches may help identify individual biomechanical profiles and inform the development and prospective evaluation of targeted rehabilitation strategies.

The limitations of this study should be considered when interpreting the results. First, the exclusive inclusion of young males limits the generalisability of the findings. Sex‐related differences have been reported in foot biomechanics, including ground reaction forces, joint moments, and joint power [79, 80]. However, female‐specific evidence in CAI remains limited, particularly regarding foot joint kinematics and kinetics during cutting tasks [53]. The findings may also not generalise to older populations, as comparable CAI studies have primarily examined young, physically active populations [14, 15, 17]. Future studies should include females and participants across broader age and activity‐level ranges. Second, the anticipated and submaximal nature of the cutting task may have reduced its sensitivity to detect between‐group differences. Third, successful return to sport was not included among the criteria used to define copers [2]. It is possible that some copers changed their sport or level of participation as a consequence of LAS. In addition, because the FAAM was not used as an inclusion criterion for CAI, self‐reported function was only moderately affected in the CAI group. Most participants with CAI and half of the copers also underwent rehabilitation, which may have further reduced between‐group differences. Fourth, part of the inter‐individual variability in kinematics and kinetics may be explained by small differences in marker placement [81]. Fifth, despite corrections to the significance threshold for ROM analyses and post hoc tests, the large number of secondary and exploratory analyses across multiple joints and planes may have increased the risk of type I error. Conversely, the absence of outcome‐specific power calculations and the use of multiplicity corrections may have increased the risk of type II error. Secondary findings should therefore be interpreted cautiously. Finally, the use of a low‐end ultrasound device may have reduced image quality and contributed to measurement variability [26].

## 5 Conclusion

In conclusion, young adults with perceived ankle instability may exhibit no or minimal differences in foot joint kinematics and kinetics during a submaximal, anticipated side‐step cutting task compared with copers and uninjured controls. Nevertheless, the lower posterior ground reaction forces observed in individuals with chronic ankle instability may reflect altered horizontal deceleration strategies. Clinical assessment and rehabilitation may therefore benefit from including change‐of‐direction tasks. Given the lower hallux flexion strength observed in individuals with chronic ankle instability and copers, exercises targeting intrinsic foot muscle function may also be considered, although their effects on cutting biomechanics and clinical outcomes require further investigation.

## Author Contributions

**Jean‐Louis Peters‐Dickie:** conceptualization, data curation, formal analysis, funding acquisition, investigation, methodology, project administration, visualization, writing – original draft, writing – review and editing. **Christine Detrembleur:** conceptualization, funding acquisition, methodology, supervision, writing – review and editing. **Sébastien Lobet:** conceptualization, funding acquisition, methodology, supervision, writing – review and editing. **Kevin Deschamps:** conceptualization, formal analysis, funding acquisition, methodology, project administration, resources, supervision, writing – review and editing.

## Funding

This work was supported by the French Community of Belgium as part of a FRIA grant (J.‐L. P.‐D.; reference number 40021590). This source of funding was not involved in the study design, data collection, analysis, and interpretation, nor in manuscript writing.

## Ethics Statement

The trial was conducted in accordance with the Declaration of Helsinki (as revised in 2013). The study was approved by the institutional ethics committee of the University Hospitals Leuven (approval number BE3222022000964) and written informed consent was obtained from all participants before data collection.

## Conflicts of Interest

The authors declare no conflicts of interest.

## Supporting information


Supporting Information S1

## Data Availability

The data that support the findings of this study are available from the corresponding author upon reasonable request.
