# Supplementary material for: Side‐Step Cutting Foot Biomechanics and Intrinsic Foot Muscle Sizes in People With Chronic Ankle Instability
Source: J Foot Ankle Res. 2026 Oct 14;19(4):e70222. doi: 10.1002/jfa2.70222 (PMC13667345; doi:10.1002/jfa2.70222)
Supplement: Supplementary file 1 [file JFA2-19-e70222-s001.docx]

Side-step cutting foot biomechanics and intrinsic foot muscle sizes in people with chronic ankle instability: SUPPLEMENTARY MATERIAL

Supplemental Table S1: Ankle sprain history, rehabilitation and use of ankle support

| Outcome | CAI | Coper | p-value |
| --- | --- | --- | --- |
| First ankle sprain | | | |
| Duration since it occurred (months) ‡ | 121.6 (104.3) | 120.4 (73.6) | 0.523 |
| Immobilization (n) | 17 yes / 5 No | 11 Yes / 13 No | 0.029* |
| Impact on weight bearing activities (days) ‡ | 15.2 (17.8) | 8.8 (13.1) | 0.145 |
| Last ankle sprain | | | |
| Duration since it occurred (months) ‡ | 30.6 (35.9) | 99.1 (72.1) | Med. diff. = 68.5 months  p < 0.001* |
| Immobilization (n) | 10 Yes / 12 No | 9 Yes / 15 No | 0.584 |
| Rehabilitation | | | |
| Pursued rehabilitation (n) | 18 Yes / 4 No | 9 Yes / 15 No | 0.002* |
| Duration (weeks) ‡ | 12.3 (14) | 6 (17) | 0.294 |
| Duration since rehabilitation started (months) † | 56.1 (37.5) | 93 (66.2) | 0.175 |
| Ankle support used during sport (n) | 6 Yes / 16 No | 0 Yes / 24 No | 0.006* |

Legend: Data are presented as counts or mean (standard deviation). Proportions were compared using Chi-squared tests, while t-test or Kruskal-Wallis tests were used for continuous data. CAI (chronic ankle instability), Med. diff. (median difference), n (number of participants), † (normality not rejected), ‡ (normality rejected), ***** (statistically significant).

Supplemental Table S2: Post-hoc tests

| Outcome | ANOVA or Kruskal-Wallis | Post-hoc tests |
| --- | --- | --- |
| Weight † | F(2,66) = 3.42,  p = 0.039* | CAI vs coper: MD = 0.38 [-5.73; 6.49], p = 1  CAI vs control: MD = 5.82 [-0.35; 11.99], p = 0.07  Coper vs control: MD = 5.44 [-0.60; 11.48], p = 0.09 |
| CAIT ‡ | H(2) = 50.0,  p < 0.001* | CAI vs coper: Med. diff. = 13, p < 0.001*  CAI vs control: Med. diff. = 13, p < 0.001*  Coper vs control: Med. diff. = 0, p = 1 |
| FAAM activities of daily living subscale ‡ | H(2) = 43.5,  p < 0.001* | CAI vs coper: Med. diff. = 5.4, p < 0.001*  CAI vs control: Med. diff. = 5.4, p < 0.001*  Coper vs control: Med. diff. = 0, p = 1 |
| FAAM sports subscale ‡ | H(2) = 50.8,  p < 0.001* | CAI vs coper: Med. diff. = 12.5, p < 0.001*  CAI vs control: Med. diff. = 12.5, p < 0.001*  Coper vs control: Med. diff. = 0, p = 1 |
| Hallux flexion strength (N·kg^-1^) † | F(2,66) = 4.58,  p = 0.014* | CAI vs coper: MD = 0.03 [-0.23; 0.27], p = 1  CAI vs control: MD = -0.25 [-0.50; 0], p = 0.050*  Coper vs control: MD = -0.28 [-0.52; -0.03], p = 0.023* |
| Posterior peak GRF (% of BW) † | F(2,65) = 4.12,  p = 0.021* | CAI vs coper: MD = -1.04 [-6.70; 4.62], p = 1  CAI vs control: MD = -6.16 [-11.87; -0.44], p = 0.03*  Coper vs control: MD = -5.11 [-10.64; 0.41], p = 0.08 |

Legend: CAI (chronic ankle instability), CAIT (Cumberland ankle instability tool), F (statistical value of the ANOVA), FAAM (foot and ankle ability measure), GRF (ground reaction force), H (statistical value of the Kruskal-Wallis test), MD (mean difference and 95% confidence interval), Med. diff. (median difference), p (p-value), † (normality not rejected), ‡ (normality rejected), * (statistically significant).

Supplemental Table S3: Side-step cutting spatiotemporal outcomes

| Outcomes | CAI | Coper | Control | p-value |
| --- | --- | --- | --- | --- |
| Speed (m·s^-1^) † | 3.40 (0.34) | 3.47 (0.32) | 3.49 (0.33) | 0.67 |
| Cadence (steps·min^-1^) † | 167.6 (12.6) | 165.4 (14.1) | 170.4 (15.7) | 0.52 |
| Step time (s) ‡ | 0.36 (0.03) | 0.36 (0.03) | 0.36 (0.03) | 0.87 |
| Step length (m) † | 1.16 (0.13) | 1.18 (0.12) | 1.17 (0.13) | 0.86 |
| Foot strike index (%) ‡ | 38.9 (16.8) | 46.5 (21.1) | 39.2 (20.5) | 0.17 |
| Foot strike pattern (n) | 7 rearfoot / 14 midfoot | 5 rearfoot / 19 midfoot | 9 rearfoot / 13 midfoot / 1 forefoot | 0.38 |
| Foot progression at foot strike, transverse plane (°) ‡ | 3.7 (7) | 5.7 (7.1) | 6.8 (9.9) | 0.18 |

Legend: Data are presented as mean and standard deviation, and numbers for categorical data. Foot strike pattern was determined using foot strike index as follows: rearfoot < 34%; midfoot 34-67%; forefoot ≥ 68% (Altman & Davis, 2012). CAI (chronic ankle instability), † (normality not rejected), ‡ (normality rejected), * (statistically significant).

Table S4: Ranges of motion and peak moment in the frontal and transverse planes

| Outcomes | CAI | Coper | Control | p-value |
| --- | --- | --- | --- | --- |
| ANKLE |  |  |  |  |
| Inv/Eve ROM: P1 (°) ‡ | 1.5 (1) | 0.9 (0.7) | 1.4 (1.3) | 0.18 |
| Inv/Eve ROM: P2 (°) ‡ | 3.6 (1.8) | 3.3 (1.9) | 3.1 (2) | 1 |
| Inv/Eve ROM: P3 (°) ‡ | 4.7 (2.4) | 4.4 (1.8) | 4.4 (1.7) | 1 |
| Abd/Add ROM: P1 (°) ‡ | 0.8 (0.7) | 1 (0.8) | 0.9 (1) | 1 |
| Abd/Add ROM: P2 (°) ‡ | 2.9 (1.6) | 2.3 (0.9) | 3.8 (2.6) | 0.62 |
| Abd/Add ROM: P3 (°) ‡ | 4.8 (2.3) | 4.3 (1.9) | 5.6 (3.1) | 0.76 |
| Inv. moment (N·m·kg-1) ‡ | 0.23 (0.18) | 0.23 (0.16) | 0.21 (0.13) | 0.91 |
| Eve. moment (N·m·kg-1) ‡ | 0.15 (0.13) | 0.15 (0.13) | 0.17 (0.13) | 0.85 |
| Add. moment (N·m·kg-1) ‡ | 0.14 (0.05) | 0.16 (0.09) | 0.14 (0.06) | 0.71 |
| Abd. moment (N·m·kg-1) ‡ | 0.17 (0.09) | 0.15 (0.11) | 0.23 (0.22) | 0.56 |
| CHOPART |  |  |  |  |
| Inv/Eve ROM: P1 (°) ‡ | 2.3 (1.2) | 2.1 (1.1) | 1.7 (1) | 0.58 |
| Inv/Eve ROM: P2 (°) † | 3.1 (1.3) | 2.7 (1.3) | 3.2 (0.9) | 1 |
| Inv/Eve ROM: P3 (°) † | 7.1 (2.1) | 7.1 (1.6) | 6.7 (1.9) | 1 |
| Abd/Add ROM: P1 (°) ‡ | 1.7 (1.1) | 1.7 (1.2) | 1.5 (1.1) | 1 |
| Abd/Add ROM: P2 (°) ‡ | 2.6 (0.9) | 2.9 (1.7) | 3.1 (1.5) | 1 |
| Abd/Add ROM: P3 (°) † | 7.5 (2.7) | 8.6 (2.1) | 8.3 (2.6) | 0.95 |
| Inv. moment (N·m·kg-1) ‡ | 0.05 (0.03) | 0.04 (0.03) | 0.06 (0.05) | 0.76 |
| Eve. moment (N·m·kg-1) ‡ | 0.49 (0.2) | 0.48 (0.15) | 0.5 (0.16) | 0.78 |
| Add. moment (N·m·kg-1) † | 0.054 (0.027) | 0.052 (0.04) | 0.052 (0.025) | 0.95 |
| Abd. moment (N·m·kg-1) † | 0.39 (0.14) | 0.37 (0.13) | 0.4 (0.2) | 0.83 |
| LISFRANC |  |  |  |  |
| Inv/Eve ROM: P1 (°) ‡ | 1 (0.7) | 0.9 (0.8) | 0.9 (0.5) | 1 |
| Inv/Eve ROM: P2 (°) † | 3.4 (1) | 3.1 (0.9) | 3.7 (1.9) | 0.82 |
| Inv/Eve ROM: P3 (°) ‡ | 6.4 (2.3) | 6.1 (2.1) | 6.9 (2.7) | 1 |
| Abd/Add ROM: P1 (°) ‡ | 1.2 (0.9) | 1 (0.6) | 0.9 (0.6) | 1 |
| Abd/Add ROM: P2 (°) † | 2.9 (1.3) | 2.8 (1) | 2.9 (1.2) | 1 |
| Abd/Add ROM: P3 (°) † | 8.4 (2.4) | 8.9 (3.2) | 8.7 (2.4) | 1 |
| Inv. moment (N·m·kg-1) ‡ | 0.058 (0.05) | 0.051 (0.045) | 0.061 (0.063) | 0.9 |
| Eve. moment (N·m·kg-1) ‡ | 0.15 (0.12) | 0.15 (0.13) | 0.17 (0.13) | 0.75 |
| Add. moment (N·m·kg-1) ‡ | 0.04 (0.019) | 0.042 (0.024) | 0.047 (0.038) | 0.99 |
| Abd. moment (N·m·kg-1) ‡ | 0.62 (0.17) | 0.65 (0.16) | 0.63 (0.17) | 0.94 |

Legend: Data are presented as mean and standard deviation. CAI (chronic ankle instability), P1, 2 and 3 (impact, active and propulsive subphases), ROM (range of motion), † (normality not rejected), ‡ (normality rejected), ***** (statistically significant). P-values related to ROM were corrected to account for the number of subphases, by multiplying the value by 3.

Table S5: Cross-correlations during side-step cutting

| Joint couple | CAI | Coper | Control | p |
| --- | --- | --- | --- | --- |
| Ankle frontal – Ankle transverse † | 0.16 (0.38) | 0.13 (0.3) | 0.27 (0.42) | 0.43 |
| Ankle frontal – Rearfoot-forefoot sagittal † | -0.14 (0.46) | 0.06 (0.4) | -0.08 (0.48) | 0.33 |
| Shank-rearfoot frontal – Rearfoot-forefoot frontal ‡ | 0.03 (0.35) | 0.08 (0.4) | 0.03 (0.41) | 0.27 |
| Ankle frontal – Rearfoot-forefoot transverse † | 0.28 (0.34) | 0.16 (0.31) | 0.24 (0.43) | 0.52 |

Legend: Data are presented as mean (standard deviation). CAI (chronic ankle instability), † (normality not rejected), ‡ (normality rejected). Cross-correlations over the stance phase were calculated between the ankle frontal-plane angle and three other foot joint angles (Deschamps et al., 2018).
Table S6: Foot muscle sizes measured with ultrasound imaging

| Measure | CAI | Coper | Control |
| --- | --- | --- | --- |
| Abductor hallucis cross sectional area (cm²) † | 2.6 (0.4) | 2.4 (0.6) | 2.3 (0.6) |
| Abductor hallucis thickness (cm) † | 1.3 (0.2) | 1.2 (0.2) | 1.1 (0.2) |
| Flexor digitorum brevis cross sectional area (cm²) † | 2.5 (0.3) | 2.3 (0.4) | 2.2 (0.5) |
| Flexor digitorum brevis thickness (cm) † | 1.1 (0.2) | 1.1 (0.1) | 1.1 (0.1) |

Legend: Data are presented as mean (standard deviation). CAI (chronic ankle instability), † (normality not rejected).

**Figure S1: Scatter plots of foot muscle sizes versus body mass index**

Scatter plots of abductor hallucis cross-sectional area (cm², left) and thickness (cm, right) versus body mass index (vertical axis).

Scatter plots of digitorum brevis cross-sectional area (cm², left) and thickness (cm, right) versus body mass index (vertical axis).

Supplemental Table S7: Correlations between intrinsic foot muscle sizes, questionnaires, and the outcomes of the physical evaluation

| Foot muscle sizes | Stat. values | Questionnaires | | | | | | Star excursion balance test | | | | Foot posture | Foot mobility | Foot strength | |
| --- | --- | --- | --- | --- | --- | --- | --- | --- | --- | --- | --- | --- | --- | --- | --- |
|  |  | CAIT ‡ | FAAM ADL ‡ | FAAM sports ‡ | FABQ PA ‡ | FABQ work ‡ | FABQ total ‡ | Anterior ‡ | Postero-medial † | Postero-lateral † | SEBT composite † | FPI-6 score ‡ | Navicular drop ‡ | Hallux strength † | Toe strength † |
| Abductor hallucis cross-sectional area  (n = 66) † | r or ρ | -0.31 | -0.33 | -0.33 | 0.07 | 0.02 | 0.07 | 0.04 | 0.09 | -0.07 | 0.01 | 0.32 | 0.07 | -0.01 | 0.03 |
|  | p | 0.011* | 0.007* | 0.006* | 0.59 | 0.86 | 0.57 | 0.76 | 0.48 | 0.60 | 0.93 | 0.009* | 0.59 | 0.96 | 0.81 |
| Abductor hallucis thickness  (n = 68) † | r or ρ | -0.33 | -0.31 | -0.26 | 0.02 | 0.05 | 0.03 | -0.07 | 0.06 | -0.09 | -0.05 | 0.33 | 0.09 | 0.01 | -0.07 |
|  | p | 0.006* | 0.010* | 0.033* | 0.85 | 0.67 | 0.82 | 0.56 | 0.65 | 0.49 | 0.68 | 0.005* | 0.48 | 0.92 | 0.55 |
| Flexor digitorum brevis cross-sectional area  (n = 68) † | r or ρ | -0.36 | -0.27 | -0.27 | 0.04 | 0.12 | 0.10 | -0.06 | -0.03 | -0.02 | -0.05 | 0.00 | -0.18 | 0.08 | 0.17 |
|  | p | 0.003* | 0.024* | 0.024* | 0.75 | 0.34 | 0.44 | 0.61 | 0.84 | 0.89 | 0.67 | 0.97 | 0.14 | 0.49 | 0.17 |
| Flexor digitorum brevis thickness  (n = 68) † | r or ρ | -0.16 | -0.15 | -0.07 | 0.03 | 0.21 | 0.13 | -0.17 | -0.12 | -0.09 | -0.18 | -0.01 | -0.07 | -0.21 | 0.00 |
|  | p | 0.20 | 0.21 | 0.58 | 0.80 | 0.09 | 0.31 | 0.17 | 0.33 | 0.46 | 0.14 | 0.94 | 0.57 | 0.09 | 0.97 |

Legend: Pearson or Spearman correlation coefficients were calculated according to the assumption of normality of the pair of variables. ADL (activities of daily living), CAI (chronic ankle instability), CAIT (Cumberland ankle instability tool), FAAM (foot and ankle ability measure), FABQ (fear avoidance belief questionnaire), FPI-6 (6-items foot posture index), n (number of participants), p (p-value), PA (physical activity), r (Pearson’s correlation coefficient), ρ (Spearman’s correlation coefficient), † (normality not rejected), ‡ (normality rejected), ***** (statistically significant).

**Figure S2: Scatter plots of foot muscle sizes versus questionnaires and foot posture**

Scatter plots of abductor hallucis cross-sectional area (horizontal axis) versus Cumberland ankle instability tool (top left), activities of daily living subscale of the foot and ankle ability measure (top right), sports subscale of the foot and ankle ability measure (bottom left), and Foot-posture index (bottom right).

Scatter plots of abductor hallucis thickness (horizontal axis) versus Cumberland ankle instability tool (top left), activities of daily living subscale of the foot and ankle ability measure (top right), sports subscale of the foot and ankle ability measure (bottom left), and Foot-posture index (bottom right).

Scatter plots of flexor digitorum cross-sectional area (horizontal axis) versus Cumberland ankle instability tool (top left), activities of daily living subscale of the foot and ankle ability measure (top right), and sports subscale of the foot and ankle ability measure (bottom).

**References**

Altman, A. R., & Davis, I. S. (2012, Feb). A kinematic method for footstrike pattern detection in barefoot and shod runners. *Gait Posture, 35*(2), 298-300. <https://doi.org/10.1016/j.gaitpost.2011.09.104>

Deschamps, K., Eerdekens, M., Geentjens, J., Santermans, L., Steurs, L., Dingenen, B., Thysen, M., & Staes, F. (2018, May). A novel approach for the detection and exploration of joint coupling patterns in the lower limb kinetic chain. *Gait Posture, 62*, 372-377. <https://doi.org/10.1016/j.gaitpost.2018.03.051>
